# Mechanical and Thermal Properties of All-Wood Biocomposites through Controllable Dissolution of Cellulose with Ionic Liquid

**DOI:** 10.3390/polym12020361

**Published:** 2020-02-06

**Authors:** Ke Chen, Weixin Xu, Yun Ding, Ping Xue, Pinghou Sheng, Hui Qiao, Suwei Wang, Yang Yu

**Affiliations:** 1 College of Mechanical and Electrical Engineering, Beijing University of Chemical Technology, Beijing 100029, China; chenke0903@outlook.com (K.C.); xueping@mail.buct.edu.cn (P.X.); wangsw90@163.com (S.W.); 2 College of Materials Science and Engineering, Beijing University of Chemical Technology, Beijing 100029, China; vanda_xu@163.com (W.X.); qiaoh@263.net (H.Q.); 3 State Key Laboratory of Bio-based Fiber Manufacturing Technology, China Textile Academy, Beijing 100025, China; shengph2000@aliyun.com; 4 School of Civil and Environmental Engineering, University of Technology Sydney, Sydney, NSW 2007, Australia; yang.yu@uts.edu.au

**Keywords:** all-wood biocomposites, ionic liquid, controllable dissolution, cellulose, mechanical properties, thermal stability

## Abstract

All-wood biocomposites were prepared with an efficient method. The ionic liquid of 1-butyl-3-methylimidazolium chloride (BMIMCl) was used to impregnate manchurian ash (MA) before hot-pressing, and the all-wood biocomposites were prepared by controllable dissolving and regenerating the cellulose in MA. The Fourier transform infrared analysis suggested that all the components of MA remained unchanged during the preparation. X-ray diffraction, thermogravimetric and scanning electron microscope analysis were carried out to study the process parameters of hot-pressing pressure and time on the crystallinity, thermal properties and microstructure of the all-wood biocomposites. The tensile strength of the prepared all-wood biocomposites reached its highest at 212.6 MPa and was increased by 239% compared with that of the original MA sample. The thermogravimetric analysis indicated that as the thermo-stability of the all-wood biocomposites increased, the mass of the residual carbon increased from 19.7% to 22.7% under a hot-pressing pressure of 10 MPa. This work provides a simple and promising pathway for the industrial application of high-performance and environmentally friendly all-wood biocomposites.

## 1. Introduction

Biocomposites, which are composed of matrix resins and natural fibers, have recently received wide attention due to their high cost-effectiveness, good environmental sustainability and potential to compete with man-made fiber-reinforced polymer composites [1,2,3,4]. Especially for the field of using bio-based polymer as a matrix resin to manufacture composite materials, more and more researchers have begun to devote themselves to the research in this field, because of the high rate of depletion of petroleum resources, and growing global environmental and social concerns [5,6,7,8]. Wood is one of the most abundant renewable resources on the earth and it should be a long-term goal to develop wood-based biocomposites to replace the petroleum-based composite materials that are not recyclable at present [9,10].

Generally, wood-based biocomposites can be divided into two types: blended wood-based biocomposites, which are composed of wood and synthetic components, and all-wood biocomposites (AWCs). AWCs are the composite materials that are composed only of wood components (cellulose, hemicellulose, lignin, etc.) and have the advantages of biodegradability and high mechanical strength. Among all wood components, cellulose is a kind of semi-crystalline polymer, whose content percentage in wood can reach up to 40%–50%. The natural cellulose is composed of a highly ordered crystalline region (cellulose I) and an amorphous region [11]. While the former can be used as the reinforcing phase to provide excellent mechanical properties for the composites, and the theoretical elastic modulus is as high as 138 GPa [12,13]. However, cellulose and other components in wood like hemicellulose and lignin are neither meltable nor soluble in water or common organic solvents [14]. This poor processability of wood limits its various industrial applications.

In the early 21st century, a new processing method was developed by Swatloski et al. to prepare the all-cellulose composites using ionic liquids [15]. The mechanical properties of the composites can be reinforced by the dissolution and regeneration of cellulose. The prepared all-cellulose composites possessed excellent interfacial compatibility, great mechanical properties and easy recyclability because of the single cellulose components [16,17]. Compared with classical biocomposites (natural fiber-reinforced composites based on either a thermoset or thermoplastic matrix), the all-cellulose composites exhibited lower density, and were completely biodegradable, combined with high strength and high stiffness. The application of ionic liquid for AWCs has also been briefly explored in producing all-wood biocomposites. Shibata et al. [18] investigated the preparation and properties of all-wood biocomposites which were produced through compressing the BMIMCl-impregnated wood flour. Nevertheless, the tensile strength and modulus of the all-wood composites (5.3 MPa and 1.9 GPa, respectively) were not improved when compared with those of the native cedar wood (20.8 MPa and 1.0 GPa, respectively). Then, they [19] also studied the properties of the lumber of hinoki cypress treated by partial dissolution in BMIMCl and hot-pressing. Although the tensile modulus of the prepared all-wood composites (6.33 GPa) had a significant improvement, the tensile strength (51 MPa) was not improved compared with that of original wood (around 63 MPa). The preparation process was also cumbersome and time-consuming. The long duration of post annealing was also against energy conservation and environmental protection. Moreover, the hot-pressing temperatures were relatively high, which may lead to the degradation of wood components.

The present study provided a low energy consumption strategy without an annealing process. The original wood of manchurian ash (MA) was used to prepare all-wood bicomposites. The processing cycle time was shorted to 6 h. Different hot-pressing temperatures and times were studied to evaluate the effects on the properties of all-wood biocomposites. The crystallinity, microstructures, mechanical and thermal properties of the obtained all-wood composites were investigated systematically compared with those of original MA sample.

## 2. Materials and Methods

### 2.1. Materials

The sheets of manchurian ash (MA) with a thickness of 4.3 mm were purchased from Shaoxing Fengsen Wood Co., Ltd. (Shaoxing, China), which were used as the sole precursor material for the preparation of all-wood biocomposites. MA was cut into sheets with size of 55 × 40 × 4.3 mm and dried in a vacuum oven at 100 °C for 24 h prior to use. 1-Butyl-3-methylimidazolium chloride (BMIMCl) with a purity of ≥95%, purchased from J&K Scientific Ltd. (Beijing, China), was used as the solvent of cellulose in MA. The melting point of BMIMCl was 73 °C. It was also dried in a vacuum oven at 100 °C for 24 h before being utilized in order to remove residual moisture. Acetonitrile with a grade of analytical reagent (purity ≥99.0%) was provided by Sinopharm Chemical Reagent Co., Ltd. (Shanghai, China), and it was used to remove BMIMCl in the composites after hot-pressing.

### 2.2. Preparation of the All-Wood Biocomposites

**Figure 1:**
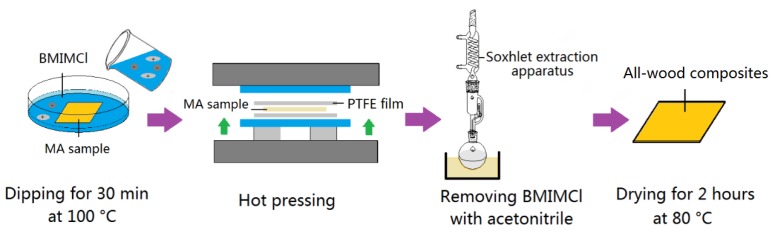
Preparation procedures of all-wood biocomposites.

The cut MA samples were immersed in the ionic liquid of BMIMCl at 100 °C for 1 h. Then, the MA samples impregnated with BMIMCl were hot-pressed at 190 °C under certain pressures and for certain times. The temperature of hot-pressing was selected as 190 °C through the exploratory experiment. When the pressing temperature was higher than that temperature, (for example, 200 °C), the sample indicated that carbonation occurred and affected the final performance of the all-wood biocomposites. When the temperature was lower than 190 °C, hot pressing would cause the sample to crack along the texture. Polytetrafluoroethylene (PTFE) films with thickness of 0.5 mm were placed on the both top and bottom of the samples to facilitate demoulding. Experiments with different hot-pressing pressures and times were carried out to study the effects on the properties of the composites. The specific processing conditions are given in Table 1. The prepared samples were named in relation to the processing condition, as shown in the table. BMIMCl in the composites was removed with acetonitrile at 90 °C for 3 h by a Soxhlet extraction apparatus. After drying for 2 h in a hot air circulating oven at 80 °C, the all-wood biocomposites were obtained. The preparation procedures are shown in Figure 1. Firstly, the influence of pressure variables on the all-wood biocomposites was studied, and it was found that the hot-pressing pressure of 10 MPa was the most suitable one. Then, the hot-pressing time was studied.

### 2.3. Characterization

#### 2.3.1. Fourier Transform Infrared (FTIR) Study

FTIR spectra measurements were performed by an infrared spectrometer of the Brooke Spectrometer Company (Tensor 27, Karlsruhe, Germany). Samples before and after BMIMCl treatment were characterized with a range of 500–4000 cm^−1^. BMIMCl and MA were also tested. The samples were pre-treated by the KBr method before testing.

#### 2.3.2. X-ray Diffraction Analysis

X-ray diffraction (XRD) analysis was performed by a X’Pert Pro X-ray diffractometer (PaNalytical Co. Ltd., Eindhoven, The Netherlands), using Cu Kα radiation (wavelength, λ = 0.154 nm) at 40 kV of power and 14 mA of current. The curve was recorded in the range of 2*θ* = 3°–40° at a scanning rate of 2.0°/min. The relative amount of crystallinity was calculated by Segal’s crystallinity index [20]. It is defined as:

(1) $$CrI=\frac{I_{002}-I_{am}}{I_{002}}\times 100\%$$

where *I*_002_ is the amplitude of the (0 0 2) diffraction peak (typical 2*θ* = 22.7°) and *I*_am_ is the amplitude of the plot at 2*θ* = 18°, which is used as an indicator of the intensity of amorphous cellulose [19].

#### 2.3.3. Scanning Electron Microscope

The morphology of the samples was observed by scanning electron microscopy (SEM), using a JSM-6360 machine (JEOL Co. Ltd., Tokyo, Japan). The surfaces and cross sections of samples were coated with platinum before SEM observation. The morphology of the fracture surface was observed after fracturing the sample in the liquid nitrogen.

#### 2.3.4. Thermal Gravimetric Analysis

Thermogravimetric analysis (TGA) tests of the samples were carried out under a nitrogen flow of 50 mL/min on a TGA instrument (Mettler Toledo TGA/DSC1, Zurich, Switzerland). The temperature range was from room temperature to 575 °C and the heating rate was 50 °C/min.

#### 2.3.5. Mechanical Properties

The tensile properties of the all-wood biocomposites were measured by using an Instron 1122 universal testing machine (5000 N, Instron Co. Ltd., Havecon, UK) at room temperature. The mechanical properties of samples in this study were measured according to ASTM D-1708 [21]. After drying, the all-wood biocomposites samples were cut into a dumbbell shape (length 55 mm, narrowest width 10 mm, thickness 0.3–0.6 mm) and the span length was 25 mm. The tensile properties of the samples were measured at a testing speed of 5 mm/min. At least five specimens per set of conditions were tested and the average and standard deviation of the tensile properties of samples were calculated.

## 3. Results and Discussion

### 3.1. FTIR Characterization of Composites Samples

**Figure 2:**
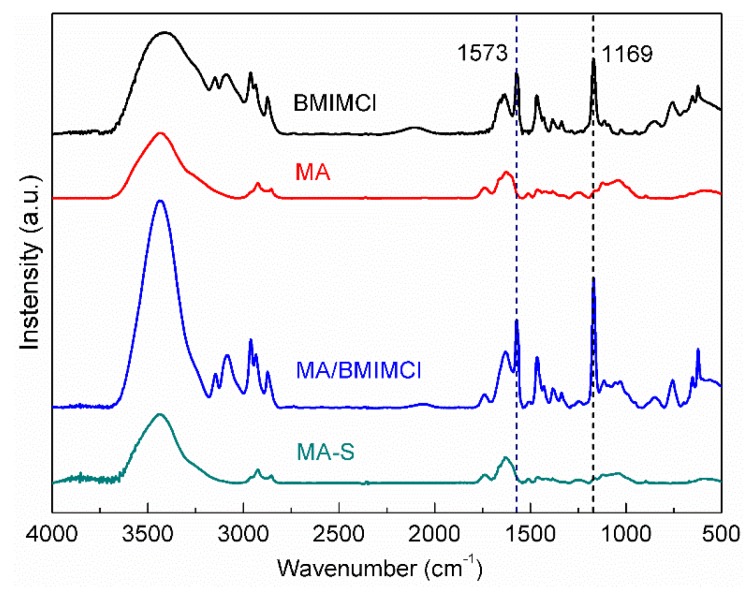
FTIR spectra of 1-butyl-3-methylimidazolium chloride (BMIMCl), manchurian ash (MA), MA impregnated with BMIMCl (MA/BMIMCl) and Soxhlet-extracted MA (MA-S).

FTIR spectra technology was carried out for the composite samples before and after extraction with acetonitrile, in order to determine whether ionic liquids existed in composites samples. BMIMCl and MA were also tested, and the results are shown in Figure 2.

Generally, MA was composed of cellulose, xylan and lignin. It can be seen from Figure 2 that MA had the characteristic bands at around 1000–1200 cm^−1^ due to the C-O stretching vibration of cellulose and xylan [19]. MA impregnated with BMIMCl (MA/BMIMCl) showed characteristic bands related to C-N stretching vibration at 1169 cm^−1^ and the imidazolidinium framework vibration at 1573 cm^−1^, which was consistent with that of BMIMCl. As for Soxhlet-extracted MA (MA-S), the characteristic bands at 1169 cm^−1^ and 1573 cm^−1^ almost disappeared, indicating that there was no more BMIMCl existing in MA-S after Soxhlet extraction. The extracted ionic liquid can be reused to dissolve cellulose in MA. Moreover, the spectra of MA and MA-S were quite similar and no new peaks appeared in the MA-S sample, which meant that there was no chemical reaction during the impregnation and extraction processes of MA.

### 3.2. Crystallinity

**Figure 3:**
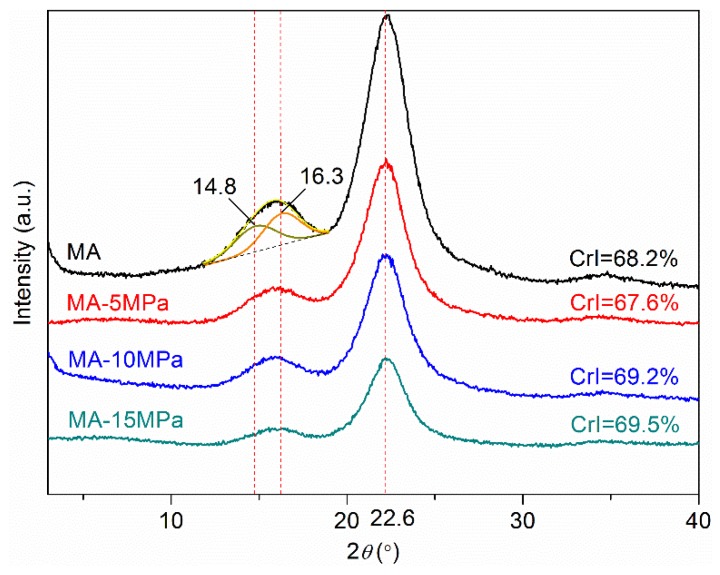
XRD profiles of the MA sample and the all-wood biocomposites with different hot-pressing pressures.

The effect of processing conditions on the crystallinity of all-wood biocomposites was discussed first. Figure 3 shows the XRD profiles of the all-wood biocomposites with different hot-pressing pressures, compared with MA. The original MA sample had the conspicuous characteristic peaks at 2*θ* = 22.6° for the (0 0 2) plane, but the characteristic peaks at 2*θ* = 13°–18.5° were not distinct enough and formed a broad peak. After peak separation treatment, it can be seen clearly that there were characteristic peaks at 2*θ* = 14.8° for the (1 0 1) plane and 2*θ* = 16.3° for the (1 0 $\bar{1}$) plane. The related coefficient R^2^ was up to 99.92%. The XRD curve of MA was consistent with the pattern of cellulose I structure. As shown in Figure 3, the peak positions of the all-wood biocomposites were the same as that of the MA sample, meaning that cellulose I structure was kept during the controllable dissolution in BMIMCl, hot-pressing and extraction process. Generally, dissolution and regeneration of cellulose I were believed to lead to a transformation to cellulose II, which was reported to be a more stable form of cellulose due to the anti-parallel packing of the single cellulose chains in contrast to the parallel packing in cellulose I [22]. In past studies, it was also revealed that the cellulose I structure was able to be maintained in the case of wood powder regenerated from wood solution in the ionic liquid [9]. In this study, the unchanged crystalline structure of cellulose I was attributed to the dissolution of only minimal amounts of cellulose. As for the crystallinity, when applied with a relatively low pressure (5 MPa), the crystallinity of all-wood biocomposites (67.6%) was lower than that of the original MA sample (68.2%), indicating that a small amount of crystalline cellulose dissolved into the BMIMCl. Then, the crystallinity of the all-wood biocomposites increased with the increasing of the hot-pressing pressure, reflecting that a considerable amount of crystalline cellulose regenerated during the hot-pressing process.

**Figure 4:**
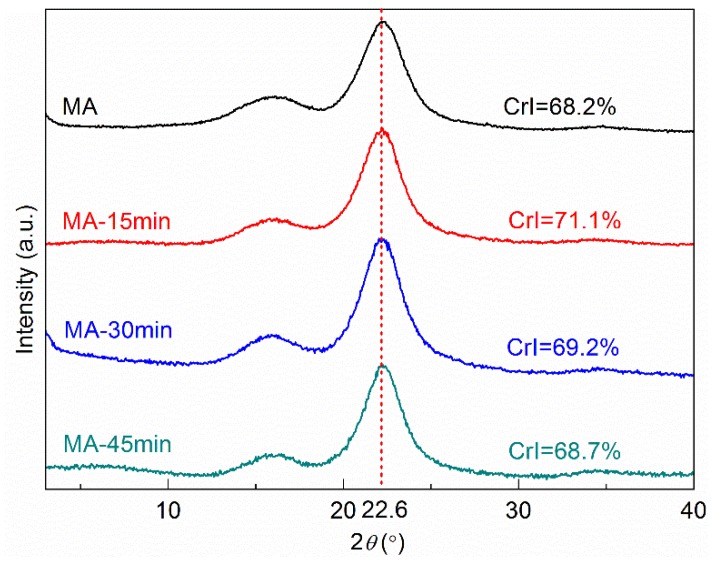
XRD profiles of the MA sample and the all-wood biocomposites with different hot-pressing times.

The effect of hot-pressing time on the crystallinity of all-wood biocomposites is presented in Figure 4. Similarly, under different hot-pressing times, the characteristic peaks of all-wood biocomposites samples did not change, indicating that the crystalline form of cellulose in the composites did not change. The crystallinity of all the all-wood biocomposites was higher than that of the MA sample. However, the crystallinity was decreased with the increasing of the hot-pressing time. The highest crystallinity of the all-wood biocomposites was present in the MA-15min sample, at 71.7%.

### 3.3. Thermal Properties

**Figure 5:**
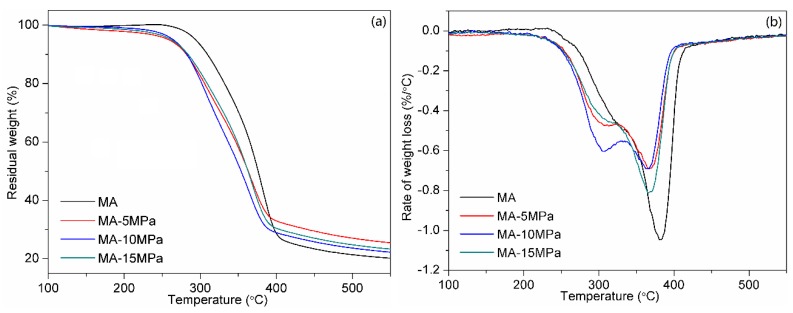
(**a**) Thermogravimetric (TG) and (**b**) derivative thermogravimetric (DTG) curves of the MA sample and the all-wood biocomposites with different hot-pressing pressures.

The effects of hot-pressing pressure and time on the thermal properties of the all-wood biocomposites were also studied. Figure 5 shows the TGA curves and the derivative thermogravimetry (DTG) curves of the MA sample and the all-wood biocomposites with different hot-pressing pressures. The degradation temperature of 5% weight loss (T_d5_) of MA (293.9 °C) was much higher than that of the all-wood biocomposites (around 261.7 °C). In previous research, it has been reported that an increase in the crystallinity of cellulose caused a rise in the thermal degradation temperature [23]. However, the fact that the MA-15MPa sample with the highest crystallinity of 69.5% had a lower T_d5_, compared with than MA-5MPa sample suggests that other factors like the dissolution effect of BMIMCl may play a more significant role. It was known that pressing the BMIMCl-impregnated cellulose at temperatures over 100 °C for a long time would cause a considerable reduction in the degree of polymerization (DP) [17,24,25]. In this study, BMIMCl not only dissolves cellulose but also lignin and xylan in MA. The decrease of T_d5_ for the all-wood biocomposites may also be attributed to the disruption of hydrogen bonds and reduction in the DP of the MA components during treatment with BMIMCl at a high hot-pressing temperature of 190 °C. In addition, the amount of residual carbon in the all-wood biocomposites was increased after hot-pressing. The amount of residual carbon in the all-wood biocomposites sample reached the highest value (24.9%) at 5 MPa. The values of the maximum degradation temperatures can be seen from the DTG curves in Figure 5b. There were two thermal degradation stages during the thermogravimetic analysis for the all-wood biocomposites samples and only one thermal degradation stage for the MA sample.

**Figure 6:**
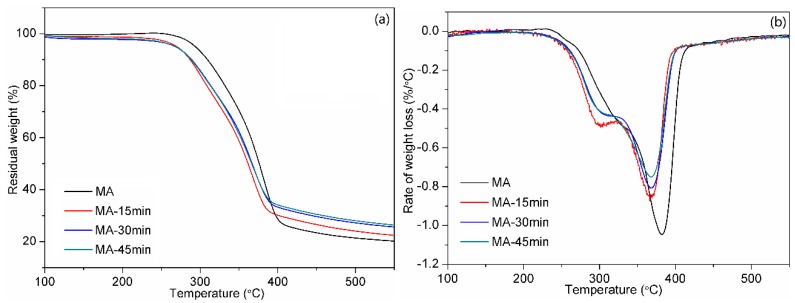
(**a**) TG and (**b**) DTG curves of the MA sample and the all-wood biocomposites with different hot-pressing temperatures.

Figure 6 presents the effect of hot-pressing time on the thermal properties of all-wood biocomposites. The T_d5_ of all-wood biocomposites with different hot-pressing pressures (around 271.2 °C) were also decreased compared with original MA sample (293.9 °C). The amount of residual carbon in the all-wood biocomposites was increased after hot-pressing and showed an increase trend with the increase in hot-pressing time. The amount of residual carbon of MA-45min sample reached the highest value as 26.4% and increased by 30% compared with the original MA sample. The maximum degradation temperatures of the all-wood biocomposites are shown in Figure 6b. It was seen that two thermal degradation stages presented during the thermogravimetic analysis for the all-wood biocomposites samples, and the maximum degradation temperatures of the all-wood biocomposites (around 367.9 °C) were decreased compared with original MA sample (382.3 °C).

### 3.4. Scanning Electron Microscopy Study of the Composites

**Figure 7:**
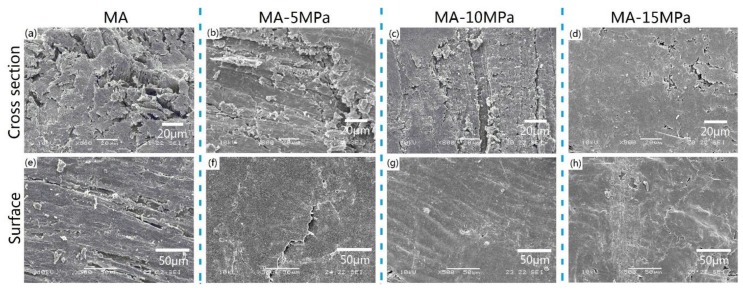
Scanning electron microscopy (SEM) micrographs of the cross section of samples: (**a**) MA, (**b**) MA-5MPa, (**c**) MA-10MPa, (**d**) MA-15MPa and SEM micrographs of the surface of samples: (**e**) MA, (**f**) MA-5MPa, (**g**) MA-10MPa, (**h**) MA-15MPa.

Scanning electron microscopy (SEM) images of both cross sections and surfaces of the all-wood biocomposites were observed. Figure 7 exhibits the effects of hot-pressing pressure on the microstructures of the all-wood biocomposites. As can be seen in Figure 7a–d, compared with the original MA sample, the materials in the cross section of the all-wood biocomposites were more dense. With the increase in hot-pressing pressure, the cross-section porosity of the all-wood biocomposites decreased gradually. However, things were different when considering the surfaces of the all-wood biocomposites with different hot-pressing pressures. Although the surfaces of the all-wood biocomposites became smoother than that of the MA sample, the smoothest surface appeared in the sample MA-10MPa. When applied with a hot-pressing pressure of 15 MPa, some cracks appeared on the surface of MA-15MPa sample, indicating that the woody tissue was somewhat broken by the excessive pressure. Therefore, from the perspective of SEM micrographs, the hot-pressing pressure of 10 MPa would be an appropriate choice for all-wood biocomposites preparation.

**Figure 8:**
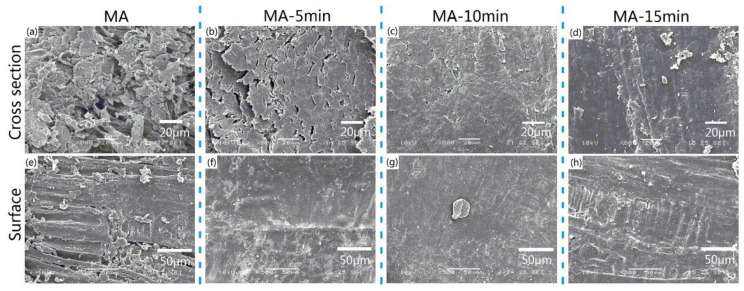
SEM micrographs of the cross section of samples: (**a**) MA, (**b**) MA-15min, (**c**) MA-30min, (**d**) MA-45min and SEM micrographs of the surface of samples: (**e**) MA, (**f**) MA-15min, (**g**) MA-30min, (**h**) MA-45min.

After the hot-pressing pressure was determined as 10 MPa, the effects of different hot-pressing times on the microstructures of the all-wood biocomposites were also studied. Figure 8a–d presents the comparison of the cross-section SEM images of the MA samples and the all-wood biocomposites with different hot-pressing times. The MA sample exhibited a microporous structure on the cross section. With the increase in hot-pressing time, the cross section of the all-wood biocomposites became more compact and the porosity decreased greatly. As for the surface microstructure of the all-wood biocomposites, when the hot-pressing time was 30 minutes, the surface of the all-wood biocomposites was the most smooth. The surface of MA-15min was not completely flat due to the short hot-pressing time, while the surface of MA-45 min was damaged by high temperature contact for a long time.

### 3.5. Mechanical Properties

**Figure 9:**
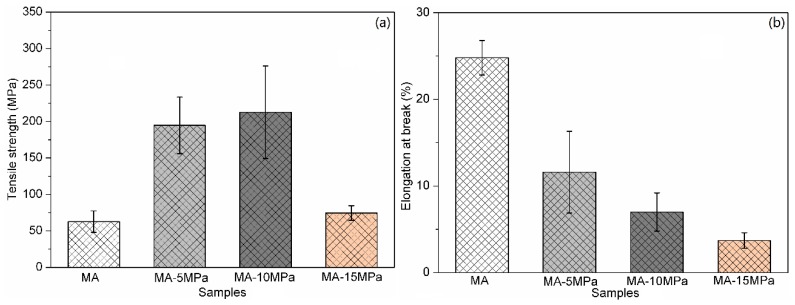
(**a**) Tensile strength and (**b**) elongation at break of the MA sample and the all-wood biocomposites with different hot-pressing pressures.

Figure 9 shows the tensile properties of MA, MA-5MPa, MA-10MPa and MA-15MPa. All the all-wood biocomposites had a higher tensile strength than that of the MA sample, but lower elongation at break. The MA-10MPa sample exhibited the highest tensile strength, reaching 212.6 MPa, and was increased by 239% compared with the MA sample. The significant decrease in the tensile strength of the MA-15MPa sample could be related to the surface cracks observed in Figure 7d,h, and then excessive pressure led to the collapse of the internal wood tissue. While the elongation at the break of the all-wood biocomposites decreased with the increasing of the hot-pressing pressure, the elongation at break of sample MA-15MPa was only 3.7%, which was 85.1% lower than the original MA sample. Compared with the MA sample, the standard deviations of tensile strength (σ_t_) of sample MA-5MPa and sample MA-10MPa were larger, while the tensile strength of sample MA-15MPa was more stable. As for the standard deviations of elongation at break (σ_e_), it showed the same trend. The detailed tensile properties of the all-wood biocomposites with different processing conditions are shown in Table 2.

**Figure 10:**
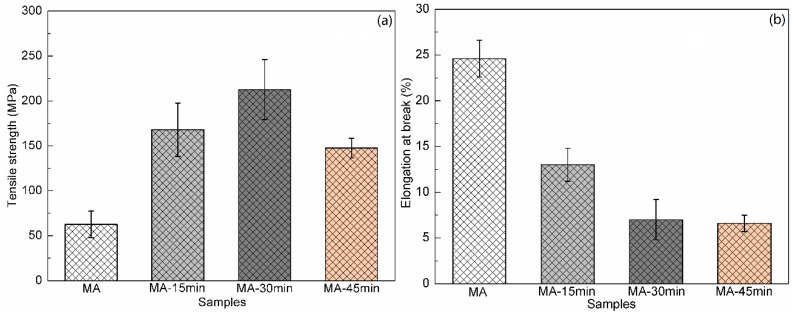
(**a**) Tensile strength and (**b**) elongation at break of MA sample and all-wood biocomposites with different hot-pressing time.

The effect of hot-pressing time on the tensile strength of all-wood biocomposites was also studied, as shown in Figure 10. When applied with a short period (15 min) of hot-pressing, the improvement in tensile strength of the all-wood biocomposites was limited. The tensile strength of the MA-30min sample reached the highest, but that of sample MA-45min was lower. That was because that long hot pressing time caused the degradation of wood tissue on the composites’ surface, as shown in Figure 8h, resulting in the reduction of tensile strength. However, this was not the same as the reduction in tensile strength under high hot-pressing pressure caused by the collapse of the internal wood tissue, so the decrease was limited. Furthermore, the elongation at break of the all-wood composite also decreased with the increasing of the hot-pressing time. According to the above test results, the optimal hot-pressing pressure and time were 10 MPa and 30 min, and the all-wood biocomposites in this study exhibited the highest tensile strength at 212.6 MPa and elongation at break of 7.0%, which can be used as the alternatives to traditional fiber reinforced composites. The effect of temperature on the stability of the tensile properties of the all-wood biocomposites was relatively weak. The tensile strength and elongation at break of the all-wood biocomposites remained relatively stable at different hot-pressing temperatures.

## 4. Conclusions

Through the controllable dissolving of the cellulose in manchurian ash (MA) with ionic liquid of BMIMCl, all-wood biocomposites were successfully manufactured in this work. With the help of this environment-friendly solvent of BMIMCl, the all-wood biocomposites were prepared with an efficient hot-pressing strategy and had excellent mechanical properties. The all-wood composites exhibited the highest tensile strength of 212.6 MPa and elongation at break of 7.0%, when hot pressing at 190 °C, 10 MPa for 30 min. The thermogravimetric analysis showed that as the thermo-stability of the all-wood biocomposites increased, the mass of residual carbon increased from 19.7% to 22.7% under a hot-pressing pressure of 10 MPa. The X-ray diffraction analysis indicated that the crystalline form of cellulose I in all-wood biocomposites remained unchanged during the controllable dissolving, hot-pressing and Soxhlet extraction process. This work provided a simple and promising pathway for the industrial application of high performance and environmentally friendly all-wood biocomposites.

## Figures and Tables

**Table 1:** Sample codes and their corresponding processing conditions.

Sample Code	Hot-Pressing Temperature (°C)	Hot-Pressing Pressure (MPa)	Hot-Pressing Time (min)
MA	0	0	0
MA-5MPa	190	5	30
MA-10MPa	190	10	30
MA-15MPa	190	15	30
MA-15min	190	10	15
MA-30min	190	10	30
MA-45min	190	10	45

**Table 2:** Tensile properties and standard deviation.

Sample Code	Tensile Strength (MPa)	σ_t_ (MPa)	Elongation at Break (%)	σ_e_ (%)
MA	62.7	14.7	24.8	2
MA-5MPa	194.8	38.8	11.6	4.7
MA-10MPa	212.6	63.4	7	2.2
MA-15MPa	74.6	9.9	3.7	0.9
MA-15min	168.0	29.6	13	1.8
MA-30min	214.1	32.8	6.7	2.4
MA-45min	147.5	11	6.6	0.9
